# The Aspartate-Semialdehyde Dehydrogenase of *Edwardsiella ictaluri* and Its Use as Balanced-Lethal System in Fish Vaccinology

**DOI:** 10.1371/journal.pone.0015944

**Published:** 2010-12-29

**Authors:** Javier Santander, Wei Xin, Zhao Yang, Roy Curtiss

**Affiliations:** 1 The Biodesign Institute, Center for Infectious Diseases and Vaccinology, Arizona State University, Tempe, Arizona, United States of America; 2 State Key Laboratory of Bioreactor Engineering, East China University of Science and Technology, Shanghai, China; Instituto Butantan, Brazil

## Abstract

*asdA* mutants of Gram-negative bacteria have an obligate requirement for diaminopimelic acid (DAP), which is an essential constituent of the peptidoglycan layer of the cell wall of these organisms. In environments deprived of DAP, i.e., animal tissues, they will undergo lysis. Deletion of the *asdA* gene has previously been exploited to develop antibiotic-sensitive strains of live attenuated recombinant bacterial vaccines. Introduction of an Asd^+^ plasmid into a Δ*asdA* mutant makes the bacterial strain plasmid-dependent. This dependence on the Asd^+^ plasmid vector creates a balanced-lethal complementation between the bacterial strain and the recombinant plasmid. *E. ictaluri* is an enteric Gram-negative fish pathogen that causes enteric septicemia in catfish. Because *E. ictaluri* is a nasal/oral invasive intracellular pathogen, this bacterium is a candidate to develop a bath/oral live recombinant attenuated *Edwardsiella* vaccine (RAEV) for the catfish aquaculture industry. As a first step to develop an antibiotic-sensitive RAEV strain, we characterized and deleted the *E. ictaluri asdA* gene. *E. ictaluri* Δ*asdA01* mutants exhibit an absolute requirement for DAP to grow. The *asdA* gene of *E. ictaluri* was complemented by the *asdA* gene from *Salmonella*. Several Asd^+^ expression vectors with different origins of replication were transformed into *E. ictaluri* Δ*asdA01*. Asd^+^ vectors were compatible with the pEI1 and pEI2 *E. ictaluri* native plasmids. The balanced-lethal system was satisfactorily evaluated in vivo. Recombinant GFP, PspA, and LcrV proteins were synthesized by *E. ictaluri* Δ*asdA01* harboring Asd^+^ plasmids. Here we constructed a balanced-lethal system, which is the first step to develop an antibiotic-sensitive RAEV for the aquaculture industry.

## Introduction

Aspartate β-semialdehyde deshydrogenase (Asd; EC 1.2.1.11), a highly conserved homodimeric enzyme encoded by the *asd* gene, is involved in the conversion of β-aspartyl phosphate to aspartate β-semialdehyde. Asd is an enzyme common to the biosynthesis of the essential amino acids lysine, threonine, methionine, and isoleucine. It also performs a key step in the production of diaminopimelic acid (DAP), a required component for the peptidoglycan synthesis of Gram-negative and some Gram-positive bacterial cell walls [1], [2], [3], [4] and an immediate precursor to lysine. *asd* mutants have an obligate requirement for DAP, and in the absence of DAP they undergo lysis. This has been demonstrated by gene-knockout studies with *Legionella pneumophila* [5], *Salmonella* Typhimurium [6] and *Streptococcus mutans* [7].

The Asd enzyme is also found in plants, where lysine is synthesized via the DAP pathway [8], [9]. In contrast, mammalian cells neither synthesize nor use DAP as a substrate in any metabolic pathway, and lysine is not synthesized since it is an essential amino acid that is obtained from dietary sources [5], [10], [11]. Also lysine, threonine, methionine, and isoleucine are essential amino acids in the diet of teleostei fish [12], [13], [14], [15], [16], [17], suggesting the absence of both the DAP/lysine synthesis pathway and Asd enzyme in fish cells.

Since DAP is absent from mammalian tissues, deletion of the *asd* gene has been exploited to develop a balanced-lethal system for vaccine delivery vehicles using a cloned *asd* gene as a selective marker in place of antibiotic-resistance markers, which are totally impractical in vivo [6]. Introduction of an Asd^+^ plasmid into *asd* mutants makes the bacterial strain plasmid-dependent. This dependence on the Asd^+^ plasmid vector creates a balanced-lethal complementation between the bacterial strain and the recombinant plasmid [18]. Asd^+^ vectors introduced into live recombinant attenuated *Salmonella* vaccines have been used to deliver heterologous antigens [19]. The construction of live attenuated recombinant bacterial vaccines not only require the absence of antibiotic-resistance markers in their recombinant plasmid, but also in their chromosomal deletions.

*Edwardsiella ictaluri,* a Gram-negative bacterial pathogen, is the cause of enteric septicemia in catfish, which causes losses estimated at $50–80 million annually [20]. The current USDA licensed vaccine, live *E. ictaluri* AQUAVAC-ESC® (Intervet Inc.), has been selected by multiple passages in increased concentrations of the antibiotic rifampicin [21], [22], [23]. The selected spontaneous mutant strain presented an attenuated phenotype missing part of the lipopolysaccharide (LPS) [20], [24]. Although there are FDA and USDA regulations against the use of antibiotic resistance in live attenuated bacterial vaccines for birds, mammals, and humans, the catfish industry currently allows antibiotic-resistant vaccine strains. Despite the fact that the current vaccine against enteric septicemia in catfish is antibiotic resistant, by using this vaccine we have learned that *E. ictaluri* live attenuated vaccines can be easily delivered to young fish and stimulate both humoral and cellular immunity of long duration [25], [26]. These results provide guidance to design live attenuated antibiotic-sensitive vaccines for the catfish aquaculture.

As a first step in developing an antibiotic-sensitive live recombinant *E. ictaluri* vaccine strain (RAEV), we adapted suicide vector technology [27] to *E. ictaluri* to construct defined unmarked chromosomal deletion mutations, for instance the *asd* deletion. Two *E. ictaluri asd* genes were identified, a functional a*sdA* and a non-functional *asdB* pseudogene. The *asdA* gene was deleted by using the described suicide vector technology. Using Asd^+^ expression vectors [19], we developed a balance-lethal system compatible with *E. ictaluri* native plasmids, to express and secrete heterologous proteins through the type II secretion system. The virulence of the *E. ictaluri* Δ*asdA* mutant, harboring an AsdA^+^ expression vector, was evaluated in vivo in the catfish (*Ictalurus punctatus*) and in the zebrafish (*Danio rerio*) host models. Here we report the first balanced-lethal vector-host system in *E. ictaluri*, a key in constructing antibiotic-sensitive live RAEV for the catfish industry.

## Results

### Sequence analysis

**Figure 1:**
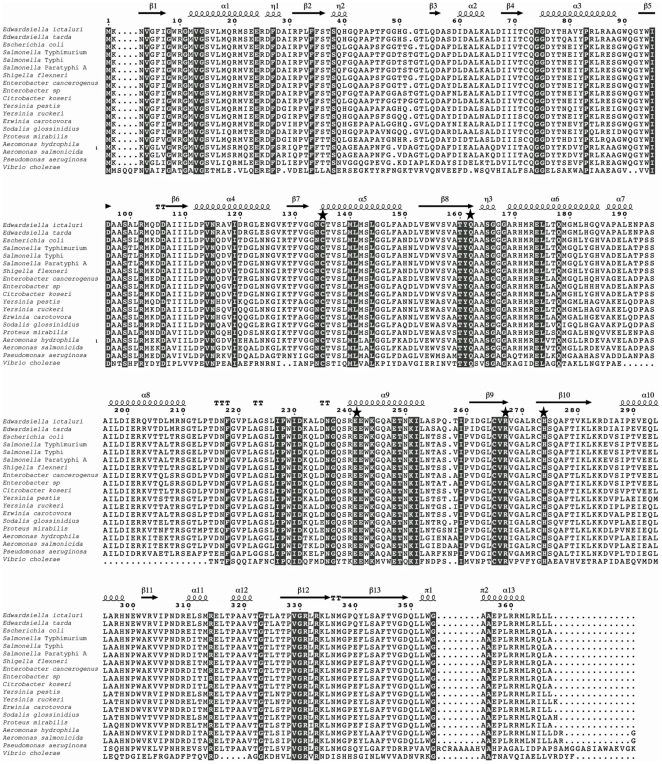
Sequence alignment among representative members of the AsdA family. The secondary structure at the top of the alignment corresponds to the *E. ictaluri* AsdA enzyme (spirals represent α-helix; arrows represent β-sheet). Conserved amino acids residues are indicated in grey. The stars indicated the key catalytic active site residues (Cys-135, Gln-162, Glu-241, Arg-267, and His-274). The AsdA sequences were obtained from NCBI's Entrez Protein database for *Edwardsiella ictaluri* YP_002935083.1; *Edwardsiella tarda* YP_003297386.1; *Escherichia coli* AP_004358.1; *Salmonella* Typhi NP_807591.1; *Salmonella* Paratyphi A YP_152515.1; *Salmonella* Typhimurium AAB69392.1; *Shigella flexnieri* YP_690789.1; *Shigella sonnei* YP_312455.1; *Citrobacter koseri* YP_001456333.1; *Enterobacter cancerogenus* ZP_05969786.1; *Enterobacter* sp. YP_001178547.1; *Yersinia pestis* NP_671174.1; *Yersinia ruckeri* ZP_04615435.1; *Proteus mirabilis* YP_002152826.1; *Aeromonas hydrophila* ABK39477.1; *Aeromonas salmonicida* YP_001142146.1; *Sodalis glossinidius* YP_456010.1; *Vibrio cholerae* YP_001217562.1; *Pseudomonas aeruginosa* NP_251807.1; *Erwinia carovora atrosepticum* YP_052242.1.

To develop a balanced-lethal system we first characterized the *asd* genes present in *E. ictaluri*. The genome of *E. ictaluri* has two *asd* gene sequences, *asdA* (gene ID 7960734) and *asdB* (gene ID 7959931). Sequence and structural alignment between functional representative bacterial Asd proteins reveals that 22 amino acid residues (∼6%) are strictly conserved out of 367 residues in *E. ictaluri* AsdA (Fig. 1). *E. ictaluri* AsdA has 28%, 81%, 82%, 84%, and 97% amino acid similarity to the Asd of *Streptococcus mutans*, *Salmonella enterica*, *Escherichia coli, Yersinia* (*Y. pestis* and *Y. ruckeri*), and *E. tarda,* respectively. The overall domain organization of *E. ictaluri* AsdA is similar to other Gram-negative Asd-family members, presenting an N-terminal domain comprising the NAD binding site and a C-terminal catalytic domain (Fig. 1). The same set of key functional groups in the active sites (Cys-135, Gln-162, Glu-241, Arg-267, and His-274) are conserved in *E. ictaluri* AsdA and likely have the same catalytic mechanism as other Asd enzymes (Fig. 1).

**Figure 2:**
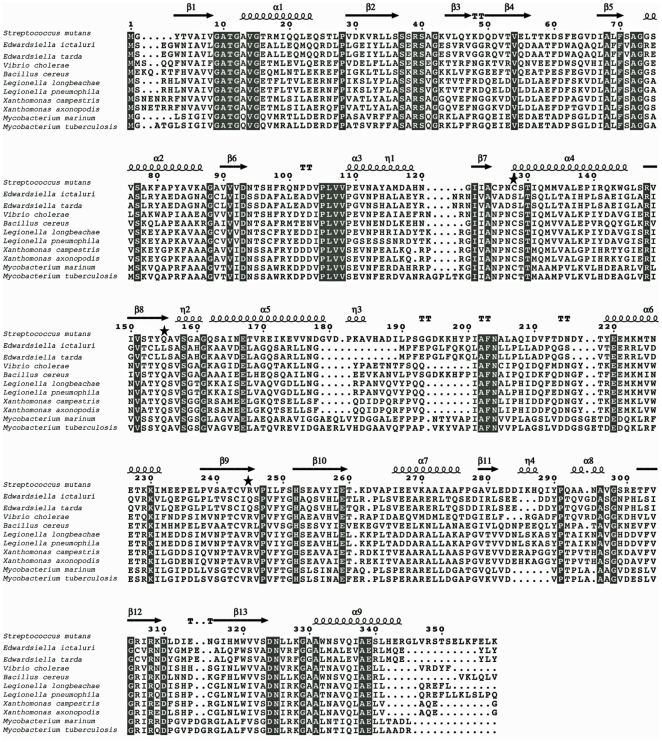
Sequence alignment among representative members of the AsdB family. The secondary structure at the top of the alignment corresponds to the *S. mutans* AsdB enzyme (spirals represent α-helix; arrows represent β-sheet). Conserved amino acids residues are indicated in grey. The stars indicated the key catalytic active site residues not present in AsdB from *Edwardsiella.* The AsdB sequences were obtained from NCBI's Entrez Protein database for *Streptococcus mutans* NP_721384.1; *Edwardsiella ictaluri* YP_002934124; *Edwardsiella tarda* YP_003296462; *Vibrio cholerae* YP_001217630.1; *Bacillus cereus* YP_085142.1; *Legionella longbeachae* CBJ10915; *Legionella pneumophila* YP_096311.1; *Xanthomonas axonopodis* NP_643032.1; *Xanthomonas campestris* NP_637897.1; *Mycobacterium tuberculosis* NP_218225.1; *Mycobacterium marinum* YP_001853481.1.

The sequence and structural alignment between representative bacterial AsdB proteins reveals that 52 amino acid residues (∼15%) are strictly conserved out of 336 in *E. ictaluri* AsdB (Fig. 2). The *E. ictaluri* AsdB has 30%, 32%, 40%, 75%, and 99% amino acid similarity to the AsdB of *Streptococcus mutans*, *Mycobacterium marinum, Vibrio cholerae, Y. pestis*, and *E. tarda,* respectively. In contrast to AsdA, the overall domain organization of *E. ictaluri* AsdB is similar to other Gram-positive Asd-family members. However, *E. ictaluri* AsdB lacks key functional groups in the active sites (Cys-135, Gln-162, and Arg-267) and likely has no catalytic activity.

The guanine plus cytosine (G+C) content found in the *E. ictaluri asdA* gene was 62%, significantly higher than the 54% of G+C found in the *Escherichia coli asdA* gene. Overall DNA comparison of the *asdA* gene showed that the *E. ictaluri asdA* gene shared 72% identity with the *Escherichia coli asdA* gene.

**Figure 3:**
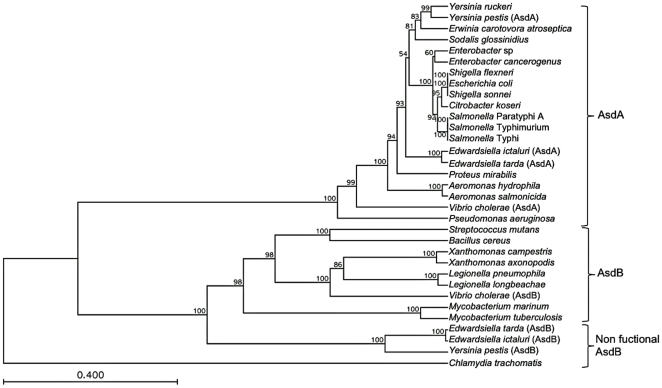
Phylogenetic tree constructed by the unweighted pair group method with arithmetic mean. Bootstrap values indicate the number of times that a given node was detected out of 100. The Asd sequences were obtained from NCBI's Entrez Protein database for *Edwardsiella ictaluri* YP_002935083.1; *Edwardsiella tarda* YP_003297386.1; *Escherichia coli* AP_004358.1; *Salmonella* Typhi NP_807591.1; *Salmonella* Paratyphi A YP_152515.1; *Salmonella* Typhimurium AAB69392.1; *Shigella flexnieri* YP_690789.1; *Shigella sonnei* YP_312455.1; *Citrobacter koseri* YP_001456333.1; *Enterobacter cancerogenus* ZP_05969786.1; *Enterobacter* sp. YP_001178547.1; *Yersinia pestis* NP_671174.1; *Yersinia ruckeri* ZP_04615435.1; *Proteus mirabilis* YP_002152826.1; *Aeromonas hydrophila* ABK39477.1; *Aeromonas salmonicida* YP_001142146.1; *Sodalis glossinidius* YP_456010.1; *Vibrio cholerae* YP_002810714.1; *Pseudomonas aeruginosa* NP_251807.1; *Erwinia carovora atrosepticum* YP_052242.1; *Streptococcus mutans* NP_721384.1; *Edwardsiella ictaluri* YP_002934124; *Edwardsiella tarda* YP_003296462; *Vibrio cholerae* YP_001217630.1; *Bacillus cereus* YP_085142.1; *Legionella longbeachae* CBJ10915; *Legionella pneumophila* YP_096311.1; *Xanthomonas axonopodis* NP_643032.1; *Xanthomonas campestris* NP_637897.1; *Mycobacterium tuberculosis* NP_218225.1; *Mycobacterium marinum* YP_001853481.1; *Chlamydia trachomatis* YP_002887982.1.

In terms of phylogeny, the bacterial Asd family is subdivided into two structural branches consisting of the enzymes from Gram-negative and Gram-positive bacteria [28] (Fig. 3). The *E. ictaluri* AsdA enzyme belongs to the Gram-negative branch, in contrast to AsdB that belongs to the Gram-positive branch (Fig. 3). *Edwardsiella* species comprise a linage that diverged from the ancestral trunk before the divergence of some other enteric bacteria, such as *Salmonella* and *Escherichia* [29], [30]. The phylogenetic position of the *E. ictaluri* AsdA enzyme corresponds with the *E. ictaluri* genome phylogenetic position (Fig. 3). Inside of the AsdB branch, a non-functional AsdB branch composed of *Edwardsiella* and *Yersinia* AsdB sequences was identified (Fig. 3), indicating that these non-functional AsdB proteins may have a common origin.

### Construction and characterization of *asdA* mutants

The construction of *E. ictaluri* Δ*asdA* mutants was performed first by using pEZ101, a pR112 (Cm) base suicide vector (Tables 1 and 2). pEZ101 was conjugated from *Escherichia coli* χ7213 to *E. ictaluri* J100 and *E. ictaluri* J102 using the methods described for *E. ictaluri* [31] and *Escherichia coli* [32]. The selection of transconjugants was carried out in BHI agar supplemented with Col, DAP, and Cm. We did not recover transconjugants by using pEZ101. Therefore, we constructed and used pEZ102, a pMEG-375 (Cm, Amp) base suicide vector (Table 2). The selection of transconjugants was carried out in BHI agar supplemented with Col, DAP, Amp or Cm. Transconjugants were recovered in the presence of Amp, but not in the presence of Cm. Transconjugants Amp^r^, harboring pEZ102 (Amp, Cm), were sensitive to Cm. We determined that *E. ictaluri* is highly sensitive to Cm. Small colonies (>0.5 mm) harboring pEZ102 were recovered in a Cm concentration below 1 µg/ml. Using BHI agar supplemented with Col, DAP, and Cm (1 µg/ml), transconjugants were not recovered using pEZ101 (Cm) or pEZ102 (Amp, Cm). Certainly, these results indicate that Cm selection and Cm-base suicide vectors are not useful to genetically manipulate *E. ictaluri*.

**Table 1:** Bacterial strains and plasmids.

Strain	Relevant characteristics	Source or reference
*Escherichia coli*		
χ6212	F^−^ Δ(*argF-lacZYA*)-*U169 glnV44* l^−^ *deoR* f80d*lacZ*Δ*M15 gyrA96 recA1 relA1 endA1* Δ*asdA4* Δ(*zhf-2*::Tn*10*) *thi-1 hsdR17*; Tet^r^	[60]
χ7213	*thr-1 leuB6 fhuA21 lacY1 glnV44 recA1* Δ*asdA4* D(*zhf-2*::Tn*10*) *thi-1* RP4-2-Tc::Mu [λ*pir*]; Km^r^	[53]
χ7232	*endA1 hsdR17 (rK-, mk+) supE44 thi-1 recA1 gyrA relA1* Δ*(lacZYA-argF) U169* λ*pir deoR (*f*80dlac*Δ*(lacZ)M15)*	Lab collection
*Edwardsiella ictaluri*		
J100	Wild-type; pEI1^+^; pEI2^+^ API20E 40040057; smooth LPS; Col^r^ DAP^+^	[59]
J102	Wild-type; pEI1^+^; pEI2^+^ API20E 40040057; smooth LPS; Col^r^ DAP^+^	ATCC 33202
J111	J102 derivative; Δ*asdA01*; pEI1^+^; pEI2^+^ API20E 40040057; smooth LPS; Col^r^ DAP^−^	This study
J112	J100 derivative; Δ*asdA01*; pEI1^+^; pEI2^+^ API20E 40040057; smooth LPS; Col^r^ DAP^−^	This study
*Salmonella enterica*		
χ3761	*S*. Typhimurium UK-1; wild-type	[19]
χ8958	*S*. Typhimurium UK-1 Δ*asdA33*	Lab collection
χ9112	*S*. Typhi ISP1820 Δ*asdA33*	Lab collection
χ9124	*S*. Typhi Ty2 Δ*asdA33*	Lab collection
*Yersinia pestis*		
χ10006	Δ*asdA12*	Lab collection

**Table 2:** Plasmid used in this study.

Plasmids	Relevant characteristics	Source or reference
pYA248	3,000 bp, contains 1,071 bp of *S. mutans asdA gene;* p15A *ori*	[18]
pYA575	5,730 bp, contains ∼1,330 bp of *S. mutans* DNA inserted between the EcoRI and HindIII sites of pBR322 plasmid, Amp, Tet, pBR *ori*	[36]
pYA3341	2595 bp, plasmid Asd^+^; pUC *ori*	[19]
pYA3493	3113 bp, plasmid Asd^+^; pBR *ori* β-lactamase signal sequence-based periplasmic N- terminal secretion plasmid	[19]
pYA3620	3169 bp, plasmid Asd^+^; pBR *ori* β-lactamase signal sequence-based periplasmic N- and C- terminal secretion plasmid	[19]
pYA3994	pBR *ori*, Asd^+^, GFP^+^ 3113 bp,	Lab collection
pYA3840	323 bp DNA encoding the LcrV in pYA3493	[38]
pYA4088	852 bp DNA encoding the α-helical region of PspA aa 3-285 in pYA3493	[39]
pRE112	5,173 bp, Cm, *sacB, oriV, oriT*	[27]
pMEG-375	8,142 bp, Cm, Amp, *lacZ*, R6K *ori, mob incP, sacR sacB*	[52]
pACYC184	4,245 bp, Tet, Cm, p15A *ori*	[54]
pEZ101	Δ*asdA01*, pR112	This study
pEZ102	Δ*asdA01*, pMEG-375	This study
pEZ140	SD-*asdA*, Cm, pACYC184	This study
pEZ142	P*_asdA_*-*asdA*, Cm, pACYC184	This study

**Figure 4:**
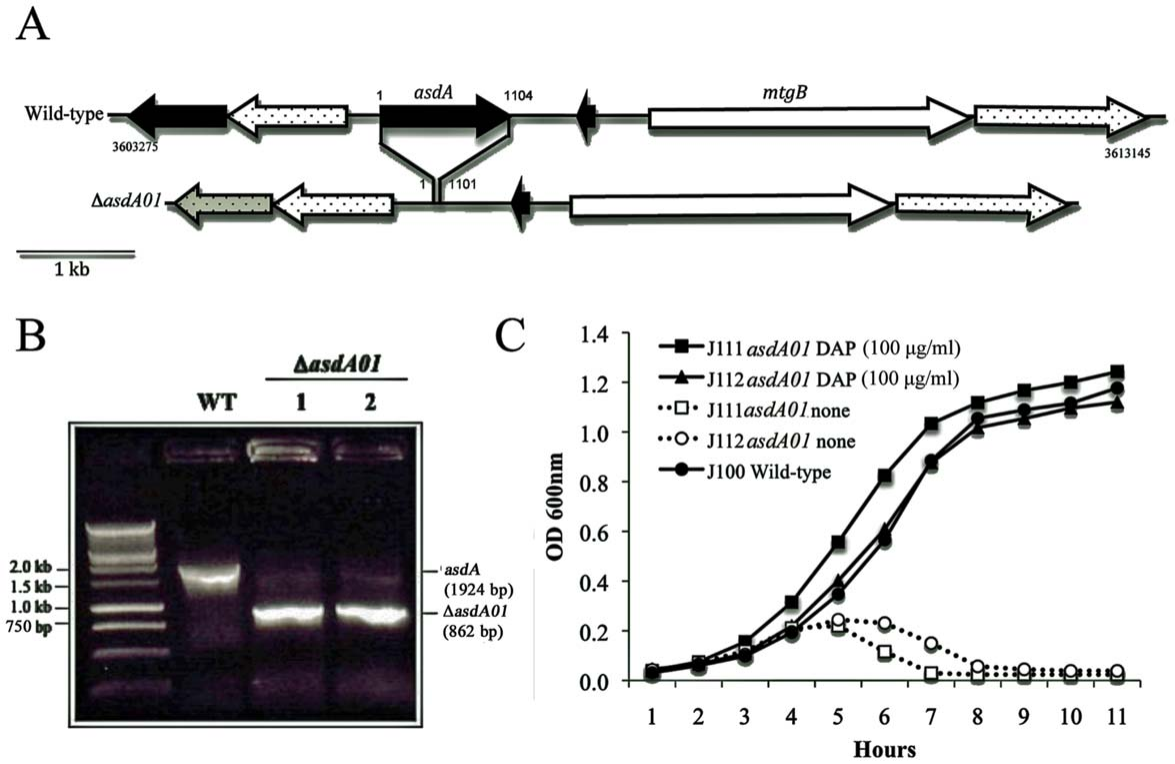
Deletion of *asdA* gene in *E. ictaluri*. **A.** Deletion map of Δ*asdA01*; **B.** Genotype verification of J112 Δ*asdA01* by PCR; **C.** Phenotype of *E. ictaluri* J111 Δ*asdA01* and J112 Δ*asdA01* mutants. The strains were grown in BHI at 28°C with agitation (180 r.p.m.).

Single colonies of *E. ictaluri* transconjugants harboring pEZ102 (Col^r^, Amp^r^), were grown in BHI, TSB or LB supplemented with DAP and Col at 28°C for 6 h with aeration (180 r.p.m.). The selection was performed in BHI, TSA and LB agar plates supplemented with DAP, Col, and 5% sucrose at 28°C for 4–5 days. BHI sucrose selection agar did not provide selection, due to *E. ictaluri* overgrowth. TSA and LB sucrose selection agar presented a satisfactory selection. Positive mutants were screened for Col^r^, Amp^s^, and DAP^–^. Several *E. ictaluri* Δ*asdA* mutants were recovered from TSA and LB sucrose-selection agar plates. The genotype was verified by PCR, and the phenotype by growth in presence of DAP and no growth in absence of DAP (Fig. 4). The biochemical profile, evaluated by API20E, did not present any difference between the wild type and Δ*asdA01* mutant strains. *E. ictaluri* strains were identified as *Edwardsiella* sp (code 4004000). These results confirmed that the AsdB present in *E. ictaluri* is non-functional, since deletion of *asdA* is enough to preclude cell growth in the absence of DAP. Thus, *asdB* can be considered a pseudo gene in *E. ictaluri*.

We evaluated reutilization of DAP by the *E. ictaluri* Δ*asdA01* mutants released from lysed Δ*asdA* cells grown in absence of DAP. Washed cells of *E. ictaluri* J112 Δ*asdA01* were diluted from 10^1^ to 10^10^ CFU/ml in BHI Col. The estimated minimum number of *E. ictaluri* Δ*asdA01* cells needed to support growth in absence of DAP was 1.3×10^8^–2.7×10^8^ CFU/ml. This is because of DAP-less death and reuse of DAP to permit growth on media without DAP.

The amount of DAP in the cell wall of *Escherichia coli* has been estimated at ∼3.5×10^6^ molecules [33]. Based on the results obtained for the minimum number of *E. ictaluri* Δ*asdA01* cells needed to support growth in the absence of DAP, and the calculated amount of DAP molecules per cell of *Escherichia coli*, we estimated that the minimum number of DAP molecules to support growth is ∼4.5×10^14^–9.5×10^14^ molecules of DAP/ml in the growth media. We evaluated the growth of *E. ictaluri* Δ*asdA01* in 10^10^ to 10^20^ molecules of DAP/ml in BHI Col. *E. ictaluri* Δ*asdA01* did not grow in concentrations below 10^14^ molecules of DAP/ml. Our previous estimation about the minimum number of DAP molecules required to support growth was confirmed, indicating that the amount of DAP in the cell wall of *E. ictaluri* is similar to *Escherichia coli*.

It has been reported that lysine, threonine, methionine, and isoleucine are essential amino acids in the diet of teleostei fish [12], [13], [14], [15], [16], [17], suggesting the absence of the DAP/lysine synthesis pathway in fish cells. We tested the growth of *E. ictaluri* J112 Δ*asdA01* in different catfish broths (1% of catfish liver, spleen, kidney and meat in BHI) in presence and absence of DAP. *E. ictaluri* J112 Δ*asdA01* was not able to grow in fish broth not supplemented with DAP. *E. ictaluri* J100 wild-type, used as control, grew in all fish broth conditions (data not shown). This result supports the idea that as mammalian cells, fish cells neither synthesize nor use DAP as substrate in any metabolic pathway.

### Complementation of *E. ictaluri asdA* gene and *E. ictaluri* Δ*asdA01* mutant

The structural analysis of *E. ictaluri* AsdA indicated that the overall domain organization is similar to other AsdA-family members and has the same set of key active-site functional groups and therefore the same catalytic mechanism as other Asd enzymes (Fig. 1). To evaluate the likely broad functionality of *E. ictaluri* AsdA enzyme, *asdA* mutants of *Escherichia coli*, *Salmonella enterica* (serovars Typhimurium, and Typhi), *Y. pestis,* and *E. ictaluri,* were complemented with the *E. ictaluri asdA* gene. Because overproduction of AsdA enzyme increases generation times [19], [34] and synthesis of Asd enzyme is proportional to the copy number of the complementing plasmid, *asdA* mutants were complemented with *E. ictaluri asdA* gene with (P_asdA-_ *asdA*) and without its promoter (SD-*asdA*), this last to decrease Asd synthesis, cloned into p15A *ori* plasmid (pACYC184; Table 2).

**Figure 5:**
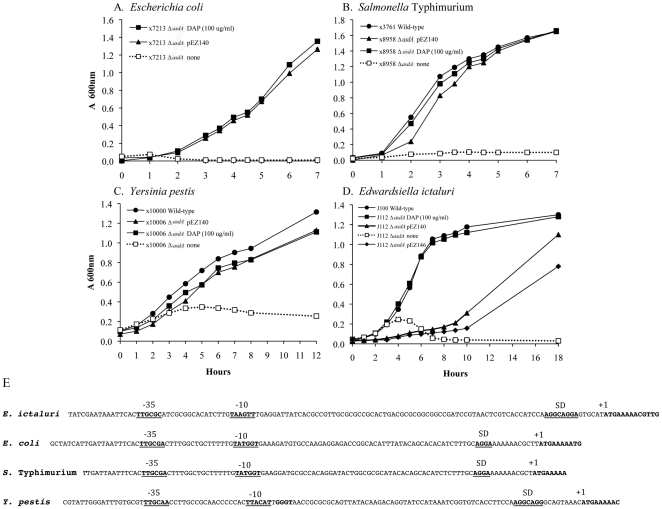
Complementation of representative Δ*asdA* mutant strains with *E. ictaluri asdA* gene. (**A–D**) Growth of representative Δ*asdA* mutant strains complemented with *asdA* from *E. ictaluri.* pEZ140 (SD-*asdA*); pEZ142 (P_asdA_-*asdA*); The strains were grown in BHI at 28°C with agitation (180 r.p.m.); (**E**) Promoter region of *asdA* gene from *E. ictaluri* and representative strains.

*Escherichia coli*, *S. enterica* and *Y. pestis* Δ*asdA* mutants complemented with *E. ictaluri* SD-*asdA* presented similar growth rates compared to wild type (Fig. 5), indicating full complementation. *E. ictaluri* Δ*asdA01* mutants complemented with SD-*asdA* presented a significantly lower growth rate than the wild type (Fig. 5). This could be due to overproduction or underproduction of Asd. It has been reported that SD-*asd* constructions do not enable Δ*asdA* strains to survive in absence of DAP if the origin of plasmid replication (*ori*) is from pSC101 or p15A. In other words, with these lower-copy-number replicons, the amount of Asd enzyme synthesized is insufficient to enable growth in absence of lysis [19]. To evaluate if the decrease in the generation time of the SD-*asdA* complemented *E. ictaluri* Δ*asdA01* strain was due to overproduction or underproduction of AsdA, complementation with P_asdA_-*asdA* (pEZ142) was performed. Complementation of *E. ictaluri* Δ*asdA01* mutants with pEZ142 decreased the growth rate even more than complementation with SD-*asdA*. These results suggest that the decreased growth rate in the *E. ictaluri* Δ*asdA01* complemented with its own *asdA* gene is due to overproduction of AsdA. There are differences in the SD regions that could justify part of the difference in the growth rate of *E. ictaluri* Δ*asdA01* complemented with its own SD-*asd* gene. The SD region of *E. ictaluri asdA* gene has an optimal spacing (6 nt) between the SD region and the ATG initiation codon of the mRNA [35] in contrast to the other bacterial species complemented with *E. ictaluri* SD-*asdA* (Fig. 5).

**Figure 6:**
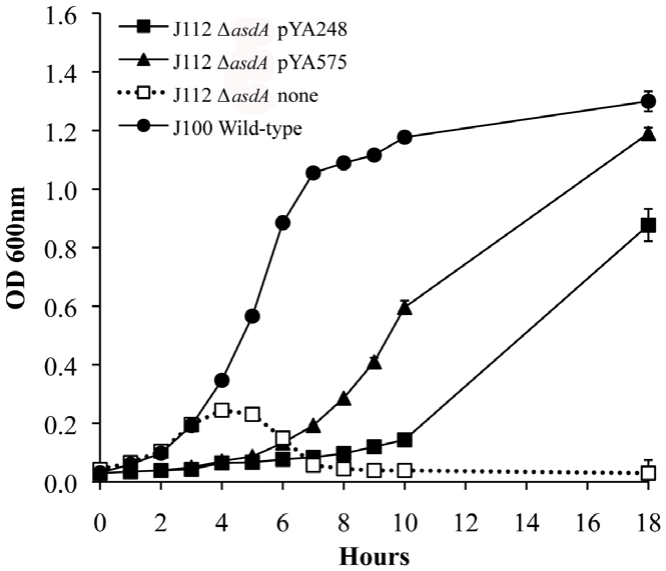
Growth of *E. ictaluri* Δ*asdA01* complemented with *asdB* from *Streptococcus mutans*. The strains were gown in BHI at 28°C with agitation (180 r.p.m.).

Complementation of *E. ictaluri* Δ*asdA01* mutants by Gram-positive AsdB enzyme was also evaluated. *Streptococcus mutans asdB* region (including the full promoter), cloned into pYA575 [36] and *S. mutans* SD-*asdB*, cloned into pYA248 [18] complemented *E. ictaluri* Δ*asdA01* mutants. However these strains presented lower growth rates than the wild type (Fig. 6). *E. ictaluri* Δ*asdA01* mutants complemented with SD-*asdB* (pYA248), presented the lowest growth rate, suggesting that *S. mutans* AsdB is probably required in higher levels to fully complement *E. ictaluri* or *S. mutans* AsdB do not interact efficiently with *E. ictaluri* aspartokinase enzymes to transfer the β-aspartyl phosphate to Asd.

### Complementation by Asd^+^ vectors to develop a balanced-lethal system in *E. ictaluri*

**Figure 7:**
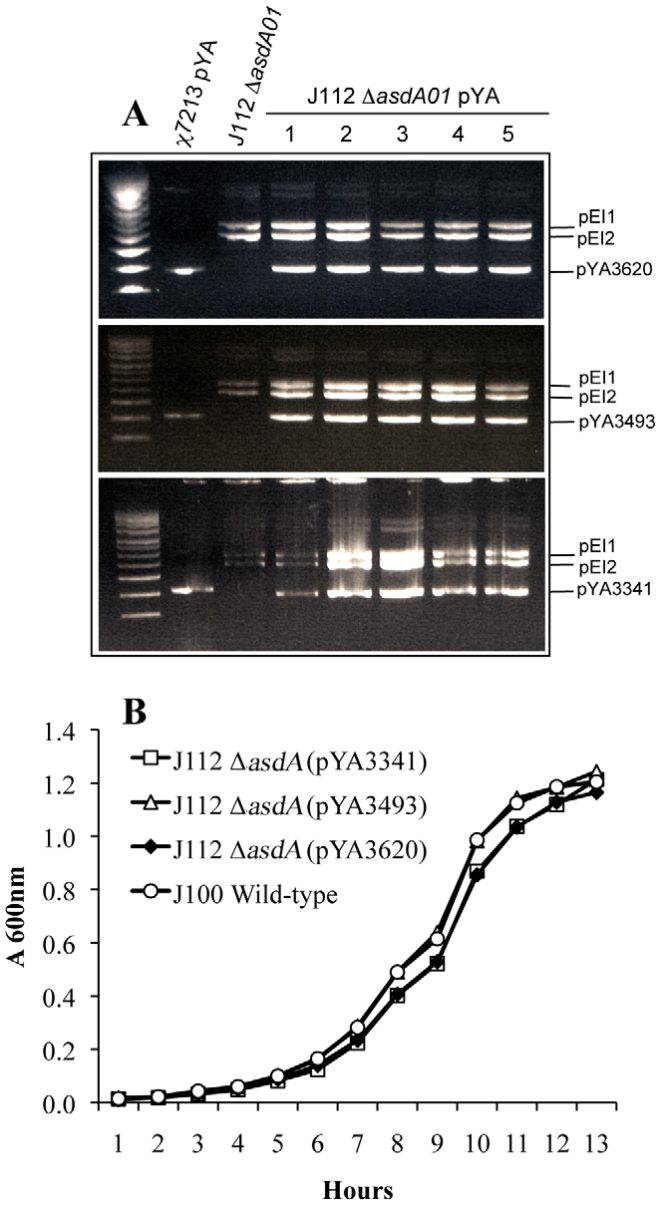
Complementation of *asdA* gene with Asd^+^ **vectors.** (**A**) Plasmid profile of *E. ictaluri* Δ*asdA01* complemented with AsdA^+^ vectors of different copy number. pEI1 (5.7 kb), pEI2 (4.9 kb), pYA3620 (3169 bp), pYA3493 (3113 bp), pYA3341 (2595 bp); Supercoiling ladder, from the top to the bottom: 16210 bp, 14174 bp, 12138 bp, 10102 bp, 8066 bp, 7045 bp, 6030 bp, 5012 bp, 3990 bp, 2972, 2067 bp; (**B**) Growth of *E. ictaluri* Δ*asdA01* complemented with different AsdA^+^ vectors; The strains were grown in BHI at 28°C with agitation (180 r.p.m.).

The *asdA* gene from *E. ictaluri* complemented *S. enterica* Δ*asdA* mutants, in addition the Asd enzymes from *E. ictaluri* and *S. enterica* share 81% similarity. Therefore, we used the Asd^+^ vectors utilized in live recombinant attenuated *Salmonella* vaccines [19] to develop a balanced-lethal system in *E. ictaluri*. The Asd^+^ vectors utilized in this study possess only the SD-*asdA* gene from *S*. Typhimurium with a modified start codon from ATG to GTG. *E. ictaluri* Δ*asdA01* mutants were complemented with the *asdA* gene from *S.* Typhimurium (Fig. 7). The growth rate of *E. ictaluri* Δ*asdA01* complemented with different copy number of Asd^+^ vectors was similar to the wild type in all cases (Fig. 7). The Asd^+^ vectors were compatible with the native plasmids of *E. ictaluri* (Fig. 7) and stable for at least 80 generations. These results show the first balanced-lethal system in *E. ictaluri*.

### Expression of genes encoding GFP protein in the AsdA^+^ vector

**Figure 8:**
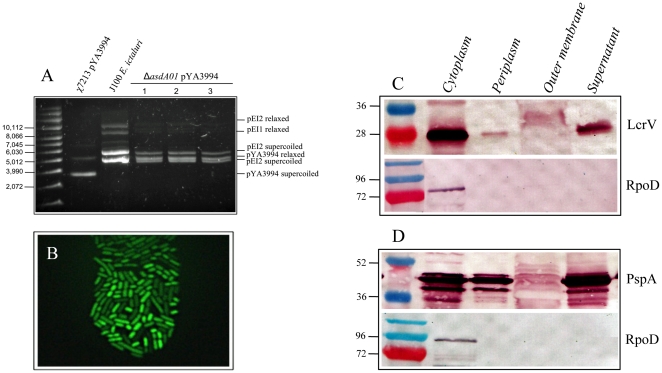
Synthesis of heterologous antigens in *E. ictaluri* J112 Δ*asdA01* by using AsdA^+^ expression vectors. **A.** Plasmid profile of J112 (pYA3994); **B.** Expression of GFP J112 (pYA3994); **C**. Expression and secretion of *Y. pestis* LcrV antigen by J112 (pYA3840); **D.** Expression and secretion of *S. pneumoniae* PspA-Rx1 antigen by J112 (pYA4088).

The synthesis of heterologous proteins, for instance GFP, cloned into Asd^+^ vectors was evaluated in *E. ictaluri* Δ*asdA01* to potentially develop live *E. ictaluri* recombinant vaccines. First, the synthesis of heterologous proteins was evaluated by using the GFP^+^ Asd^+^ vector pYA3994 (Table 2). *E. ictaluri* Δ*asdA01* mutant strains harboring the GFP^+^ Asd^+^ vector grew in absence of DAP and synthesized GFP^+^ as expected (Fig. 8). The GFP^+^ Asd^+^ vector was compatible with the native plasmids of *E. ictaluri* in the relaxed conformation (Fig. 8). The GFP^+^ Asd^+^ vector was stable in *E. ictaluri* Δ*asdA01* strains for at least 80 generations. The expression of LcrV and PspA heterologous proteins using AsdA^+^ vectors was also evaluated (see below).

### Secretion of heterologous proteins

Secretion of the heterologous antigens by live attenuated recombinant bacterial vaccines has been shown to enhance immunogenicity against the heterologous antigen [37]. The synthesis and secretion of heterologous proteins was evaluated by using the proteins derived from Gram-positive and Gram-negative bacterial strains. PspA-Rx1 from *Streptococcus pneumoniae* was utilized as a Gram-positive representative and LcrV from *Yersinia pestis* was utilized as a Gram-negative representative. The heterologous antigens, PspA-Rx1 and LcrV fused to β-lactamase signal sequence, were expressed from the Asd^+^ vectors pYA4088 and pYA3841, respectively (Table 2). Both heterologous proteins were secreted through the type II secretion system. No difference in the growth rate was observed between the recombinant *E. ictaluri* and the wild-type strain J100.

### Virulence of *E. ictaluri* Δ*asdA01* strain complemented with the AsdA*^+^* plasmid vector in catfish host and zebrafish host models

The idea to develop a balanced-lethal system in a pathogenic bacterial strain is to synthesize heterologous antigens, without the use of antibiotic-resistant genes, in either the plasmid or in the bacterial chromosome. This is the first step towards developing live recombinant bacterial vaccines. The ideal balanced-lethal system should present nearly the same level of virulence as the wild-type strain with regard to invasion and colonization of lymphoid tissues. We evaluated the virulence of *E. ictaluri* Δ*asdA01* mutants with and without the balanced-lethal system in the catfish and zebrafish hosts (Tables 3 and 4). We used pYA3493 AsdA^+^ since this vector has been used successfully in live recombinant *Salmonella* vaccines [19], [38], [39], [40]. *E. ictaluri* Δ*asdA01* was attenuated at the high dose of 10^8^ CFU, but still produced some mortality in catfish (Table 4). *E. ictaluri* Δ*asdA01* at a high dose (10^8^ CFU) was not attenuated in zebrafish and all the fish died (Table 3). However, at lower doses (10^7^-10^4^) *E. ictaluri* Δ*asdA01* was totally attenuated in zebrafish (Table 3). *E. ictaluri* Δ*asdA01* harboring the Asd^+^ vector pYA3493 increased the LD_50_ one log-fold, from 10^4^ CFU to 10^5^ CFU in orally infected catfish, and two log-fold, from 10^3^ CFU to 10^5^ CFU, in zebrafish (Tables 3 and 4). Catfish i.p. infected with *E. ictaluri* Δ*asdA01* harboring the Asd^+^ vector pYA3493 presented the same level of virulence as *E. ictaluri* wild type (Table 4). From moribund orally infected catfish, *E. ictaluri* Δ*asdA* (pYA3493) AsdA^+^ was recovered from the head-kidney, spleen and liver, indicating that *E. ictaluri* Δ*asdA* (pYA3493) AsdA^+^ colonized these lymphoid tissues.

**Table 3:** Survival of zebrafish (*D. rerio*) infected with wild-type and *E. ictaluri* Δ*asdA01* with and without Asd^+^ vectors.

	Experiment #1i.m.		Experiment #2i.m.	
E. ictaluri strains	Dose (CFU/ml)	Survivors/Total	Dose (CFU/ml)	Survivors/Total
J100 wild-type	1.5×10^8^	0/25	1.2×10^7^	0/25
	1.5×10^6^	0/25	1.2×10^3^	13/25
	1.5×10^4^	2/25	1.2×10^2^	22/25
J112 ΔasdA01	3.0×10^8^	0/10	1.7×10^7^	1/5
			1.7×10^6^	10/10
			1.7×10^4^	10/10
J112 ΔasdA01 (pYA3493)	2.1×10^8^	0/10	1.8×10^8^	0/5
	2.1×10^6^	0/10	1.8×0^6^	0/5
	2.1×10^4^	10/10	1.8×10^4^	5/5
BSG (Control)	None	10/10	None	5/5

The zebrafish were infected i.m. with 10 µl of the respective *E. ictaluri* strain.

**Table 4:** Survival of catfish (*I. punctatus*) infected with *E. ictaluri* wild type and *E. ictaluri* Δ*asdA01* with and without Asd^+^ vectors.

	Experiment #1i.p.		Experiment #2Oral	
*E. ictaluri* strains	Dose (CFU/ml)	Survivors/Total	Dose (CFU/ml)	Survivors/Total
J100 wild-type	1.5×10^8^	0/6	1.2×10^8^	1/7
	1.5×10^6^	0/6	1.2×10^6^	2/7
	1.5×10^4^	1/6	1.2×10^4^	4/7
J112 *ΔasdA01*	3.0×10^8^	3/7*	1.7×10^8^	7/8*
J112 *ΔasdA01* (pYA3493)	2.1×10^8^	0/6	1.8×10^8^	3/7
	2.1×10^6^	2/6	1.8×0^6^	4/7
	2.1×10^4^	2/6	1.8×10^4^	5/7
BSG (Control)	None	6/6	None	6/6

The catfish were infected i.p. with 100 µl and orally with 20 µl of the respective *E. ictaluri* strain.

*death within 48 h.

## Discussion

To develop a balanced-lethal system in *E. ictaluri*, we first characterized the *asdA* and *asdB* genes present in the genome of *E. ictaluri* (Fig. 1). Deletion of the *asdA* gene precluded the growth of *E. ictaluri* in absence of DAP (Fig. 4), indicating that *asdB* does not encode for a functional protein related to DAP synthesis. This is consistent with the bioinformatic analysis (Fig. 2), which showed that the AsdB enzyme lacked several key amino acid residues at the catalytic active site.

The phylogeny of Asd has two branches, AsdA related with Gram-negatives and AsdB related with Gram-positives [28]. We found a particular group of non-functional AsdB genes in *Edwardsiella* and *Yersinia*. The common origin of AsdB in these bacteria suggests that the genes might have lost their activity through evolution, and that *asdB* could be considered a pseudogene in *Edwardsiella* and *Y. pestis*.

Suicide vector technology has been successfully used in several enteric bacteria to develop antibiotic-sensitive mutants [27]. Using this technology it was possible to construct defined deletion mutations in the absence of antibiotic-resistance markers for the first time in *E. ictaluri* (Fig. 3). During this process, we determined that *E. ictaluri* is extremely sensitive to Cm, even in the presence of the *cat* gene. The *cat* gene confers high-level resistance to Cm in most bacterial species. It codes for an enzyme called chloramphenicol acetyltransferase which inactivates Cm by covalently linking one or two acetyl groups, derived from acetyl-S-coenzyme A, to the hydroxyl groups on the chloramphenicol molecule [41]. This might indicate that chloramphenicol acetyltransferase is not functional or inefficient in *E. ictaluri*. Further studies are required to answer this.

The current live attenuated *E. ictaluri* vaccine is a rifampicin-resistant strain [22]. Antibiotic resistance in live attenuated bacterial vaccines present a threat to both the animal and to human health, due to the horizontal transmission of genes, in this case by transduction. Recently lytic bacteriophages have been isolated from catfish ponds against *E. ictaluri* [42]. This suggests that temperate phages for *E. ictaluri* that can establish lysogeny might be present in these environments and could spread rifampicin resistance to native environmental bacterial flora. Here we have described a methodology to genetically engineer *E. ictaluri* without the use of antibiotic-resistance genes in the final strain. This advancement opens up the field of *E. ictaluri* live attenuated vaccine development and will provide opportunities for further research into the pathogenesis of this important organism.

Although, *E. ictaluri* Δ*asdA01* is complemented with its own *asdA* gene, the complemented strain did not grow at the same rate as the parental wild-type strain, presenting a higher growth rate. To achieve the right amount of native AsdA in *E. ictaluri* using Asd^+^ vectors requires further studies. However, *E. ictaluri* Δ*asdA01* was fully complemented by the *Salmonella* SD-*asdA* gene, allowing the development of a balanced-lethal system.

One of the major difficulties in the construction of a balanced-lethal system in *E. ictaluri* is the incompatibility of the Asd^+^ vectors with cryptic plasmids present in the bacterial strain. *E. ictaluri* possesses two native autonomous small plasmids, pEI1 and pEI2 [43], that have been implicated in virulence [44]. The Asd^+^ expression vectors were compatible with pEI1 and pEI2 native plasmids of *E. ictaluri*, indicating that the origin of replication of these plasmids, ColE1 *ori* and ColE2 *ori*-like, respectively [43], are compatible with p15A ori, pBR *ori* and pUC *ori*.

*E. ictaluri* was described by Hawke in 1979 [45], and recently sequenced (NCBI's Entrez Genome database NC_012779). Most of its genes encode for putative functions. *E. ictaluri* possesses the machinery for the type II secretion system in its genome. Therefore we evaluated the secretion of proteins by using a β-lactamase signal sequence at the N-terminal end of a recombinant protein [19], a signal required for a protein to be secreted through the system mentioned above. Recombinant proteins, cloned in the AsdA^+^ vector and using the β-lactamase signal sequence, were secreted in a similar fashion (Fig. 8) as for a *Salmonella* recombinant vaccine [37], suggesting that the type II secretion system in *E. ictaluri* is fully functional.

*Salmonella* Δ*asdA* mutants are totally attenuated in mice orally infected with 10^8^ CFU per dose [19]. *E. ictaluri* Δ*asdA01* mutants were not fully attenuated in catfish i.p. or orally infected (Table 4). Zebrafish i.m. infected with doses of 10^8^ CFU succumbed to *E. ictaluri* Δ*asdA01* mutant infection (Table 3). Lower doses of *E. ictaluri* Δ*asdA01* mutants (10^6^-10^4^ CFU) were totally attenuated (Table 3). It has been reported that *E. ictaluri* contain toxins, like hemolysin [46], [47]. We believe that the mortality caused by *E. ictaluri* Δ*asdA01* mutants, either in catfish or zebrafish, is due to a toxic shock-like effect caused by the toxins realized after this DAP dependent mutant lyse in vivo. These toxins probably are not LPS related, since fish and amphibians are resistant to the toxic effects of LPS [48], [49]. *E. ictaluri* Δ*asdA01* (pAsdA^+^) was attenuated by one log-fold in catfish animal host model (orally infected), and two log-fold in zebrafish. The next step in the construction and design of a live recombinant *E. ictaluri* vaccine is the attenuation of the bacterial strain without altering colonization of lymphoid tissues and immunogenicity. From moribund orally infected catfish, *E. ictaluri* Δ*asdA* (pAsdA^+^) were recovered from the head kidney, spleen and liver, indicating that *E. ictaluri asdA* (pAsdA^+^) colonize lymphoid tissues. The increase in attenuation in catfish orally infected with *E. ictaluri* Δ*asdA* (pAsdA^+^) could be used together with other genetic modifications to attenuate *E. ictaluri* in regard to constructing a live RAEV.

In summary, we have described methods to genetically engineer *E. ictaluri* without the use of antibiotic-resistant genes in the final strain. This opens up the field of RAEV development and will provide opportunities for further research into *E. ictaluri* pathogenesis. We have developed an antibiotic-sensitive recombinant *E. ictaluri* strain, using suicide vector technology [27] and Asd^+^ expression vectors [19]. This first balanced-lethal vector-host system in *E. ictaluri* is key in constructing antibiotic-sensitive live RAEV for the catfish industry.

## Materials and Methods

### Ethics statement

All research involving fish was conducted as per Protocol #09-1042R, approved by the Arizona State University Institutional Animal Care and Use Committee.

### Bacterial strains, plasmids, media, and reagents

The bacterial strains and plasmids are listed in Table 1 and 2. Bacteriological media and components are from Difco (Franklin Lakes, NJ). Antibiotics and reagents are from Sigma (St. Louis, MO). LB broth (tryptone, 10 g; yeast extract 5 g; NaCl 10 g; dextrose 1 g, ddH_2_O 1 L) [50], Bacto-Brain Heart Infusion broth (BHI), and Trypticase Soy Broth (TSB), were used routinely. When required, the media were supplemented with 1.5% agar, 5% sucrose, colistin sulphate (Col; 12.5 µg/ml), ampicillin (Amp; 100 µg/ml), chloramphenicol (Cm; 25 µg/ml), or kanamycin (Km; 50 µg/ml). Fish broths were prepared with fresh homogenized catfish tissues (liver, spleen, kidney, and meat; catfish specific pathogen free were from University of Arkansas at Pine Bluff) to 1% in BHI and filter sterilized (0.22 µm). Bacterial growth was monitored spectrophotometrically and/or by plating. Oligonucleotides were from IDT (Coralville, IA). Restriction endonucleases were from New England Biolabs. Taq DNA polymerase (New England Biolabs) was used in all PCR tests. Qiagen products (Hilden, Germany) were used to isolate plasmid DNA, gel-purify fragments or purify PCR products. T4 ligase, T4 DNA polymerase and shrimp alkaline phosphatase (SAP) were from Promega.

### Sequence analysis

Nucleotide Basic Local Alignment Search Tool (BLAST) was performed based on the sequences of the putative *asd* genes present in the genome sequence of *E. ictaluri* 93-146 accessed from NCBI's Entrez Genome database (NC_012779).

Asd sequences used were obtained from NCBI's Entrez Protein database. Amino acid sequence alignments were performed using the CLC Free Workbench software tool (v. 6.1 CLC bio A/S, Aarhus, Denmark). Protein structural-based alignments were performed by using the web-based interface for ESPript v.2.2 located at http://espript.ibcp.fr/ESPript/cgi-bin/ESPript.cgi [51]. Phylogenetic position of *E. ictaluri* AsdA protein was performed with CLC Free Workbench version using the unweighted pair group method with arithmetic mean (UPGMA). Bootstrap analysis was performed with 100 resamplings.

### Construction and characterization of *asdA* mutants

The recombinant suicide vector pEZ102 (Table 2) carrying the linked flanking regions (5′ 361 bp and 3′ 422 bp) to generate an in-frame deletion of the *asdA* gene was constructed as described in [52]. The Δ*asdA01* defined deletion mutation encompasses a 1,104 base pair deletion including the ATG start codon but not including the TAG stop codon. Primers (primer 1) 5′- ACATGCATGCAATGCCGTCAACGCCGCAGAAT-‘3 and (primer 2) 5′- CCGCTCGAGATGCACTCCTGCCTTGGATGGTGA -‘3 were designed to amplify the upstream *asdA* flanking region (361 bp). A *SphI* site was included in the primer 1 (underlined) and a *XhoI* site was included in primer 2 (underlined). The downstream *asdA* flanking region (422 bp) was amplified by primers (primer 3) 5′- CCGCTCGAGTGAGGCTACTGCTCTAGCCCGTGC -‘3 and (primer 4) 5′- TCGTCTAGAGCCAGATAGATTTGATGTTGTCTCTGCTGC -‘3. A *XhoI* site was included in primer 3 (underlined) and *XbaI* site was included in primer 4. The flanking regions were amplified from *E. ictaluri* J100, ligated, cloned into pRE112 and pMEG-375, and then digested with *SphI* and *XbaI*. The resulting plasmids were designated pEZ101 and pEZ102, respectively. To construct the *E. ictaluri* Δ*asdA01* mutant, the suicide plasmid was conjugationally transferred from *Escherichia coli* χ7213 [53] to *E. ictaluri* wild-type strains J100 and J102. Strains containing single-crossover plasmid insertions (*E*. *ictaluri asdA*::pEZ102) were isolated on BHI agar plates containing Col, Amp, and DAP. Loss of the suicide vector after the second recombination between homologous regions (i.e., allelic exchange) was selected by using the *sacB*-based sucrose sensitivity counter-selection system [27]. The colonies were screened for Amp^s^, Col^r^ and for growth only in presence of DAP. DAP^–^ colonies were screened by PCR using primer 1 and 4. Biochemical profiles of *E. ictaluri* strains were determined using the API 20E system (bioMériux, Marcy I'Etoile, France).

### Complementation of *asdA* gene

The *asdA* gene of *E. ictaluri*, with and without its promoter, was cloned into a pAYCY184 vector [54] by inactivating the Tet cassette at the *BamHI* and *XbaI* restriction sites. The primers used to amplify *asdA* with its promoter (P_asdA_-*asdA*) were 5′ – TCGTCTAGATCTTGTAAGTTTGAGGATTA – 3′ (upstream) and 5′ – CGGGATCCTCAGCATGCGGCGCAACGGCTC – 3′ (downstream). An *XbaI a*nd *BamHI* site were included in these primers, respectively (underlined). To amplify the *E. ictaluri* Shine-Dalgarno (SD)-*asdA* promoter-less the upstream primer 5′ – TCGTCTAGAAGGCAGGAGTGCATATGAAAAA – 3′ was used with the downstream primer previously described. An *XbaI* site was included in this primer (underlined). The *E. ictaluri* promoter-less *asdA* includes the SD AGGA region, 6 bp upstream from the ATG start codon (SD-*asdA*). The resulting plasmids, pEZ140 (SD-*asdA*) and pEZ146 (P_asd_-*asdA*) were used to complement different Δ*asdA* mutant strains. Also *asd* from *Streptococcus mutans*, cloned into pYA575 [36] and pYA248 [18], was used to evaluate complementation of *E. ictaluri* Δ*asdA01* mutants.

To create a balanced-lethal system in *E. ictaluri*, several Asd^+^ expression vectors harboring the SD-*asdA* gene sequence from *Salmonella* Typhimurium UK-1 with different origins of replication, (Table 2) [19] were transformed into *E. ictaluri* Δ*asdA01* to evaluate their complementation and stability. The growth rate of the complementing strains was evaluated in the absence of DAP. Plasmid stability was evaluated for fifty generations as described by Konjufca et al. [55].

### Expression of heterologous antigens by *E. ictaluri* Δ*asdA01*

Asd^+^ expression vectors encoding different heterologous proteins (Table 2) were transformed into *E. ictaluri* Δ*asdA01* to evaluate the expression and secretion of foreign proteins. First, the green fluorescent protein (GFP) was used to evaluate protein synthesis in the *E. ictaluri* Δ*asdA01* strain. The vector pYA3994 AsdA^+^ GFP^+^ without a peptide secretion signal sequence was transformed into *E. ictaluri* Δ*asdA01* (Table 2). The synthesis of GFP was evaluated by fluorescent microscopy. The synthesis of LcrV and PspA was evaluated by western blot and the secretion was evaluated by subcellular fractionation [37].

### Western blot analysis

To evaluate the synthesis of heterologus proteins by *E. ictaluri*, the strains were grown in 3 ml of BHI at 28°C with aeration (180 r.p.m.). The samples were collected when the culture reached the absorbance of 1.0 (O.D_600_ 1.0∼1×10^8^ cfu/ml). One ml of culture was collected and prepared for Western blot analysis [56]. The total proteins were normalized by using a nanodrop spectrophotometer (ND-1000, NanoDrop) at 25 mg/µl and separated by 10% (wt/vol) sodium dodecyl sulfate (SDS)-polyacrylamide gel electrophoresis and transferred onto nitrocellulose membranes [56]. Fat-free milk powder solution (5%, wt/vol) in PBS supplemented with 0.05% of Tween 20 (PBS-T) was used for blocking. The membrane was incubated individually with a primary mouse anti-RpoD monoclonal antibody (1∶1,000) (Neoclone), rabbit anti-LcrV polyclonal antibody (1∶1,000) [57], or rabbit anti-PspA polyclonal antibody (1∶10,000) [40], for 1 h at room temperature, washed three times with PBS-T, and then incubated with a 1∶10,000 dilution of alkaline phosphatase-conjugated anti-mouse immunoglobulin G (IgG) (Sigma) or anti-rabbit immunoglobulin G (IgG) (Sigma). Color was developed with nitroblue tetrazolium and 5-bromo-4-chloro-3-indolylphosphate (BCIP) (Amaresco).

### *Edwardsiella* subcellular fractionation

Cultures were grown in BHI at 28°C static to an OD_600_ of 0.6 and centrifuged at 5,865 g for 10 min. Periplasmic fractions were prepared by a modification of the lysozyme-osmotic shock method [58] as previously described [37]. The supernatant fluid was saved for analysis of secreted proteins. Equal volumes of periplasmic, cytoplasmic, and supernatant fractions and total lysate samples were separated by SDS-PAGE for western blot analysis.

### Determination of LD_50_ in zebrafish animal host

Zebrafish infections were performed by the methodology described by Petri-Hanson et al. [59] with modifications. The temperature of the water was 26±1°C and the fish were acclimated during 2 weeks prior to the start of the experimentation. Adult zebrafish (average weight, 0.5 g) were sedated in 100 mg/L tricaine methanesulfonate (MS-222, Sigma) and then injected intramuscularly (i.m.). Groups of zebrafish (typically 15 fish per group) were injected i.m. with 10 µl of the bacterial suspension (10^3^–10^9^ CFU) into each fish. A 3/10-cc U-100 ultrafine insulin syringe with a 0.5-in.-long (ca. 1-cm-long) 29-gauge needle (catalog no. BD-309301; VWR) was used to inject the fish. Two sets of controls were used: fish that were injected with 10 µl of sterile phosphate-buffered saline containing 0.01% gelatin (BSG) [60] and fish that were not injected. Moribund fish demonstrating clinical signs were euthanized, necropsied, and bacteria isolated as previously described [59]. The fish were fed twice daily with TetraMin Tropical Fish Flake Feed. During the experiments, the fish were observed daily, and every other day water quality was monitored for pH and NO_2_ with standard kits. The LD_50_ was calculated by the method of Reed-Muench [61]. Fish care and use was performed in accordance with the requirements of the Arizona State University, Institutional Animal Care and Use Committee.

### Determination of LD_50_ in catfish animal host

Specific-pathogen-free channel catfish (*Ictalurus punctatus*) fingerlings were used with a mean weight of 18.5±1.3 g. The animals were randomly assigned to treatment groups of 6 to 8 fish each in 100 liter tanks. Each tank was equipped with a self-contained, recirculating, biofiltered, mechanical filtered, and U.V. water treated system with 12 h of illumination daily. The water temperature was 28±1°C during the 2 weeks of acclimatization and during the experiments. The fish were fed daily with commercial Aquamax grower 400 (Purina Mills Inc., St. Louis, MO). During the experiments, the fish were observed daily, and every other day water quality was monitored for pH and NO_2_ with standards kits. Catfish were infected with 10^3^ to 10^9^ CFU of *E. ictaluri* strains (fish were not fed until 1 h after infection) orally and intra peritoneal (i.p.). The fish were anesthetized with tricaine methanesulfonate (MS-222, Sigma; 100 mg/L of water) prior to handling. The LD_50_ was calculated by the method of Reed-Muench [61]. Moribund animals were necropsied to evaluate presence of *E. ictaluri* in kidney, spleen and liver. Fish care and use was performed in accordance with the requirements of the Arizona State University, Institutional Animal Care and Use Committee.

### Bacteria preparation

Bacterial strains were grown overnight in standing cultures that were diluted 1∶20 in prewarmed BHI broth and grown with mild aeration (180 r.p.m.) at 28°C to an OD_600_ of 0.8 to 0.9 (∼10^8^ CFU/ml). Bacteria were sedimented 10 min by centrifugation (5,865 g) at room temperature and resuspended in BSG [60] to densities appropriate for the inoculation.
